# *Axl* expression is increased in early stages of left ventricular remodeling in an animal model with pressure-overload

**DOI:** 10.1371/journal.pone.0217926

**Published:** 2019-06-10

**Authors:** Montserrat Batlle, Nadia Castillo, Anna Alcarraz, Sebastian Sarvari, Gemma Sangüesa, Helena Cristóbal, Pablo García de Frutos, Marta Sitges, Lluis Mont, Eduard Guasch

**Affiliations:** 1 Institut d’Investigacions Biomèdiques August Pi i Sunyer (IDIBAPS), Barcelona, Catalonia, Spain; 2 Centro de Investigación Biomédica en Red de Enfermedades CardioVasculares (CIBERCV), Instituto de Carlos III, Madrid, Spain; 3 Institut Clínic Cardiovascular (ICCV), Hospital Clínic, Universitat de Barcelona, Catalonia, Spain; 4 Departament de Mort i Prol·liferació Cel·lular, Institut de Investigacions Biomediques de Barcelona (IIBB), Barcelona, Spain; Scuola Superiore Sant'Anna, ITALY

## Abstract

**Background:**

AXL is a receptor tyrosine kinase that has been related to kidney and vascular disorders. Heart failure patients with reduced ejection fraction have higher AXL in serum than controls. No information about Axl expression with HF progression is available.

**Methods:**

Thoracic transverse aortic constriction (TAC) was successfully performed on male Wistar rats (n = 25) with different constriction levels. Controls underwent sham surgery (n = 12). Echocardiography measurements were performed 4–8 weeks after surgery. Collagen deposition was measured with picrosirius red staining. *Axl* mRNA levels in left ventricle (LV), left kidney (LK) and ascending aorta (aAo) and the LV expression of cardiac remodeling and fibrogenic factors were quantified with real-time PCR. AXL LV protein levels were quantified with western blot and localization was analyzed by immunohistochemistry. Soluble AXL levels in plasma were assayed with ELISA.

**Results:**

Successful TAC rats were classified into LV hypertrophy (LVH) or heart failure (HF), modeling the progressive cardiac changes after pressure overload. Collagen deposition was increased only in the HF group. LV *Axl* mRNA levels were higher in LVH and HF than in Sham rats, and correlated with LVHi, and hypertrophic and fibrogenic mediators. However, no association was found with LV systolic function. AXL was expressed in LV myocytes and other cell types. Concentration of circulating sAXL in plasma was increased in the LVH group compared to Sham and HF rats. *Axl* mRNA levels were similar in all groups in the LK and aAo.

**Conclusions:**

*Axl* expression pattern suggests a role in the early progression of LV remodeling in HF but not in the later systolic dysfunction. The higher levels of circulating AXL found in HF patients most probably shed from the heart.

## 1. Introduction

AXL is a receptor tyrosine kinase that belongs to the TAM family (Tyro3, Axl, MerTK receptors). Gas6 is the only recognized Axl receptor ligand, and its binding triggers Axl oligomerization and activation of downstream signal cascades that are involved in cancer, chronic immune disorders and other diseases [1,2].

The Gas6/AXL axis regulates collagen deposition in different organs. Angiotensin II (AngII), a main pro-fibrotic factor, triggers Axl expression in VSMCs *in vitro* [3]. In the liver, Gas6/Axl promotes collagen deposition through modulation of the myofibroblast phenotype of hepatic stellate cells [4,5]. In *Axl*(-/-) mice, CCl4-induced liver fibrogenesis was blunted in comparison with wild type mice [5]. Soluble AXL (sAXL) increases in patients with liver cirrhosis [6,7], evolving as a potential biomarker for hepatopathies. Tissue-wise, *AXL* expression is increased in keloid fibroblasts [8] and in lung fibroblasts in idiophatic pulmonary fibrosis [9].

In the kidney, the receptor AXL and its ligand Gas6 have been reported to play an important role in several types of kidney disease animal models [10]. Furthermore, increased *Axl* expression has been detected in kidney samples from humans with diverse inflammatory renal diseases while barely detectable in normal kidney [11].

Evolving evidence supports that AXL plays a role in cardiovascular diseases through modulation of vascular smooth muscle cell (VSMC) phenotype [1]. *AXL* mediates the vascular response to mechanical and chemical insults and hypertension [12–14] and contributes to vascular calcification [15].

More recently, *Axl* expression in murine cardiac tissue has been demonstrated [16] and a putative role of *Axl* in heart failure (HF) is under study on grounds of experimental and clinical data. *Gas6*(-/-) mice lacking the Axl-ligand Gas6 are protected from pressure-overload left ventricle (LV) remodeling and fibrosis, while LV remodeling was remarkably increased in mice with Gas6 cardiac overexpression [17]. In humans, sAXL evolves as an attractive biomarker for HF and other cardiac conditions [18,19].

Our group previously reported higher cardiac and circulating AXL levels in HF patients with reduced ejection fraction (HFREF) than in controls, and sAXL levels predicted worse HF outcomes [20]. Nevertheless, it remains unknown the timeline of *Axl* expression in HF progression. In the present work, we aimed to analyze *Axl* expression in LV along with cardiac remodeling progression triggered by pressure-overload in a HF animal model. We also aimed to map the putative origin of the increased sAXL in HF patients by quantifying *Axl* mRNA expression in other potential origins.

## 2. Methods

### 2.1 Thoracic transverse aortic constriction (TAC)

The experimental protocols followed the European Community Directive (2010/63/UE) and Spanish guidelines (RD 53/2013) for the use of experimental animals and were approved by the Animal Research Ethics Committee of the University of Barcelona and by the Generalitat de Catalunya (protocol number 7028).

Male wistar rats (300-350g) were purchased at Charles River (Germany) and housed at the University of Barcelona animal facility in a controlled environment (12h of light/ 12h of dark) and with food and water *ad libitum*.

Thoracic transverse aortic constriction (TAC) surgery was performed in 33 rats under surgical lens. Rats were anesthesized with 2–3% inhaled isofluorane, intubated and ventilated (CWE, Ardmore, PA, USA), and kept at 37.0±0.3°C during the whole experiment with a homeothermic pad (Kent Scientific, Torrington, CT, USA). Intramuscular buprenorphine (0.03–0.05 mg/Kg) was used as analgesic. A portion of the aorta between the brachiocephalic trunk and the left carotid artery was isolated through medial suprasternal partial thoracotomy. A bended and blunted 18G or 20G needle was placed next to the aorta. Afterwards a piece of 5/0 nylon suture was placed and knot around both the vessel and needle to restrict blood flow. The needle was subsequently carefully removed. Sham rats (n = 12) were littermate controls that underwent the same surgery, but no needle and no constriction were applied. The chest cavity and the skin were carefully closed with sutures. After recovery from anesthesia, rats were placed back to their cages with food and water access *ad libitum*.

### 2.2 Electrocardiogram (ECG)

Four to eight weeks after surgery, a one-lead (DII) surface ECG was recorded. Rats were maintained at 37.0±0.3°C with a heating pad (KentScientific, Torrington, CT, USA) under inhaled isofluorane 1.5%. Recordings were obtained with PowerLab and Labchart v8.0 (AD Instruments, Oxford, UK). ECGs were analyzed offline (Labchart v8.0, AD Instruments, Oxford, UK) by an investigator blind to group assignment. During a stable 2-minute interval, every two-hundred beats were averaged and semi-automatically measured. Afterwards, the ECG parameters were manually corrected, and average results were obtained for each animal. For each rat, heart rate (HR), RR interval, P duration, P amplitude, PR interval, QRS duration, R amplitude, T amplitude, QT interval and Mitchell-corrected QT interval [21] were measured.

### 2.3 Echocardiography

Subsequent to the ECG recording, a transthoracic echocardiography was obtained under inhaled isofluorane (2–3%). Rats were placed in a heating pad and a phased-array probe 10S (4.5–11.5 MHz) was used attached to the Vivid system (GE Vingmed Ultrasound, Horten, Norway). Images were analyzed using commercially available software (EchoPac v. 108.1.6, GE Healthcare, Madrid, Spain). Left atrial and ventricle diameters, and LV functional measurements were quantified in a longitudinal parasternal plane. LV diameters at both end-diastole (LVEDD) and end-systole (LVESD) were measured at the level of the papillary muscles. The posterior wall (PW) and interventricular septum (IVS) thickness were measured at end-diastole and were indexed by the rat body weight at the sacrifice (BW). LV ejection fraction (LVEF), fractional shortening (FS), and LV mass were estimated using previously validated formulas in rodents [22,23].

Left ventricle mass was body weight-indexed to calculate a LV hypertrophy index (LVHi). Two different stages of pressure-overload were identified according to echocardiographic parameters: compensated LV hypertrophy (LVH), and overt LV systolic dysfunction and heart failure (HF).

Because of the strain and age variability of normality values in rats, and the lack of robust reference values in the literature, we established internally valid thresholds by characterizing sham rats. The fractional shortening (FS) lower limit of normality was established at the lowest value in the sham group, and the LVHi upper limit of normality was established at the highest value in the sham group. Sham-based thresholds are shown in the S1 Fig. Accordingly, those rats in the TAC group with a FS<35.2% were considered to have systolic function impairment, and those TAC rats with LVHi>3.32 g/kg were considered to have LV hypertrophy (LVH).

### 2.4 Euthanasia and sample collection

Rats were sacrificed with an overdose of 4–5% inhaled isoflurane. Blood samples were collected with EDTA tubes and centrifuged (2000 g, 10 min at 4°C). The supernatant was collected and aliquoted. The ventricular apex was cut and fixed with formol and latter embedded with paraffin. Left ventricle (LV) free wall, left kidney (LK) and ascending Aorta (aAo) tissues were extracted, minced and immediately frozen with liquid nitrogen. All samples were kept at -80°C until RNA or protein extraction.

### 2.5 Fibrosis quantification

Four-micron thick sections from the apex were stained with Picrosirius-red as described in Sanz-de la Garza et al. [24]. Six representative pictures were taken from each sample with an Olympus BX41TF light microscope and a DP73 camera (10X objective). Relative intramyocardial fibrosis was quantified blinded to group assignment, using Fiji for Windows [25]. Care was taken to exclude perivascular and endocardial and epicardial fibrosis.

### 2.6 RNA isolation and real time PCR assays

Messenger RNA extraction from LV, LK and aAo was performed with Trizol Reagent (Invitrogen, Thermo Fisher) tissue disruption followed with silica column purification following the *mir*Vana miRNA Isolation Kit protocol (Invitrogen). Total RNA (1 μg for LV and LK; 0.5 μg for aAo) was retrotranscripted with the High-Capacity cDNA Reverse Transcription Kit (Thermo Fisher). *Axl*, LV remodelling and fibrosis markers mRNA levels were measured with Real time PCR with the enzyme SYBR Select Master Mix (4472908, Thermo Fisher). Real time PCR results were normalized to the cyclophilin B (*Ppib*) housekeeping gene, and presented with the 2^-ΔΔCt^ method.

To further explore the hypertrophic remodeling processes involved in this model, we used the R384 “Cardiac hypertrophy” (Bio-Rad) PCR array including 96-well predesigned assays (assayed genes in S1 Methods) for n = 10 Sham, n = 7 LVH and n = 11 HF rats. Five μg of total RNA were retrotranscribed with the iScript cDNA Synthesis Kit (1708891, Bio-Rad). The cDNA was diluted to a final 25 ng/μl concentration and gene expression levels were assayed with the SsoAdvanced Universal SYBR Green Supermix (1725272, Bio-Rad) and the plates. Cardiac hypertrophy mRNA levels were normalized to the geometric mean of *Gapdh* and *Hprt1*, as suggested by the manufacturer. Results are shown with the 2^-ΔΔCt^ method.

A detailed protocol is provided in the S1 Methods.

### 2.7 AXL protein levels quantification in plasma and cardiac tissue

Soluble AXL (sAXL) was quantified in plasma samples with the commercial ELISA Kit for AXL Receptor Tyrosine Kinase in Rat (EKU02655, Biomatik).

Total protein extract was obtained from frozen LV samples of the Sham (n = 5), LVH (n = 8) and HF (n = 10) groups. Thirty micrograms of total protein were run in an electrophoresis setup, transferred onto a nitrocellulose membrane, checked by Ponceau staining, and incubated with a primary polyclonal anti-*Axl* antibody (ab-72069, abcam) and a goat anti-rabbit secondary antibody. Bands were scanned in Image Quant LAS4000 (GE Healthcare, OH, USA), quantified by Image Lab (Biorad) and normalized with the Ponceau staining.

A detailed protocol is provided in the S2 Methods.

### 2.8 Immunohistochemistry

Immunohistochemistry staining for AXL was performed in paraffin embedded apex slides by using the polyclonal rabbit Axl antibody (PA5-23254, ThermoFisher). A detailed protocol is provided in the S3 Methods.

### 2.9 Statistical analysis

Results are presented as mean±SEM or as percentages. Gaussian distribution was checked with Q-Q plots. For mRNA, the 2^-ΔΔCt^ results did not follow a normal distribution and a log-transformation was applied. Comparisons between the three groups (Sham, LVH and HF) were performed with a one-way ANOVA, followed by LSD tests in the case of a significant omnibus test. For the PCR array, those genes in which amplification was not achieved in >30% of samples, were excluded. Values were back-transformed to 2^-ΔΔCt^ for visual representation. Correlations were analyzed with a Pearson test; a log-transformation was performed if any variable was non-normally distributed.

To identify correlated (co-expressed) genes, a principal component analysis was performed. The ΔCt value of all genes in the cardiac hypertrophy R384 array and *Axl* mRNA levels were jointly analyzed. Missing values (n = 49 for the whole panel) were imputated with the *pca* function in R. A principal component analysis was conducted (*prcomp* function in R), and genes were plotted on the basis of their two main components.

Statistical analyses were performed with Graphpad v5 (GraphPad Software, La Jolla California USA), SPSS v23 (IBM Corp, Armonk, NY, USA) and R v3.5.2 (R Foundation for Statistical Computing, Vienna, Austria). A p-value <0.05 was considered to be statistically significant.

## 3. Results

### 3.1 TAC as a progressive HF model

**Fig 1:**
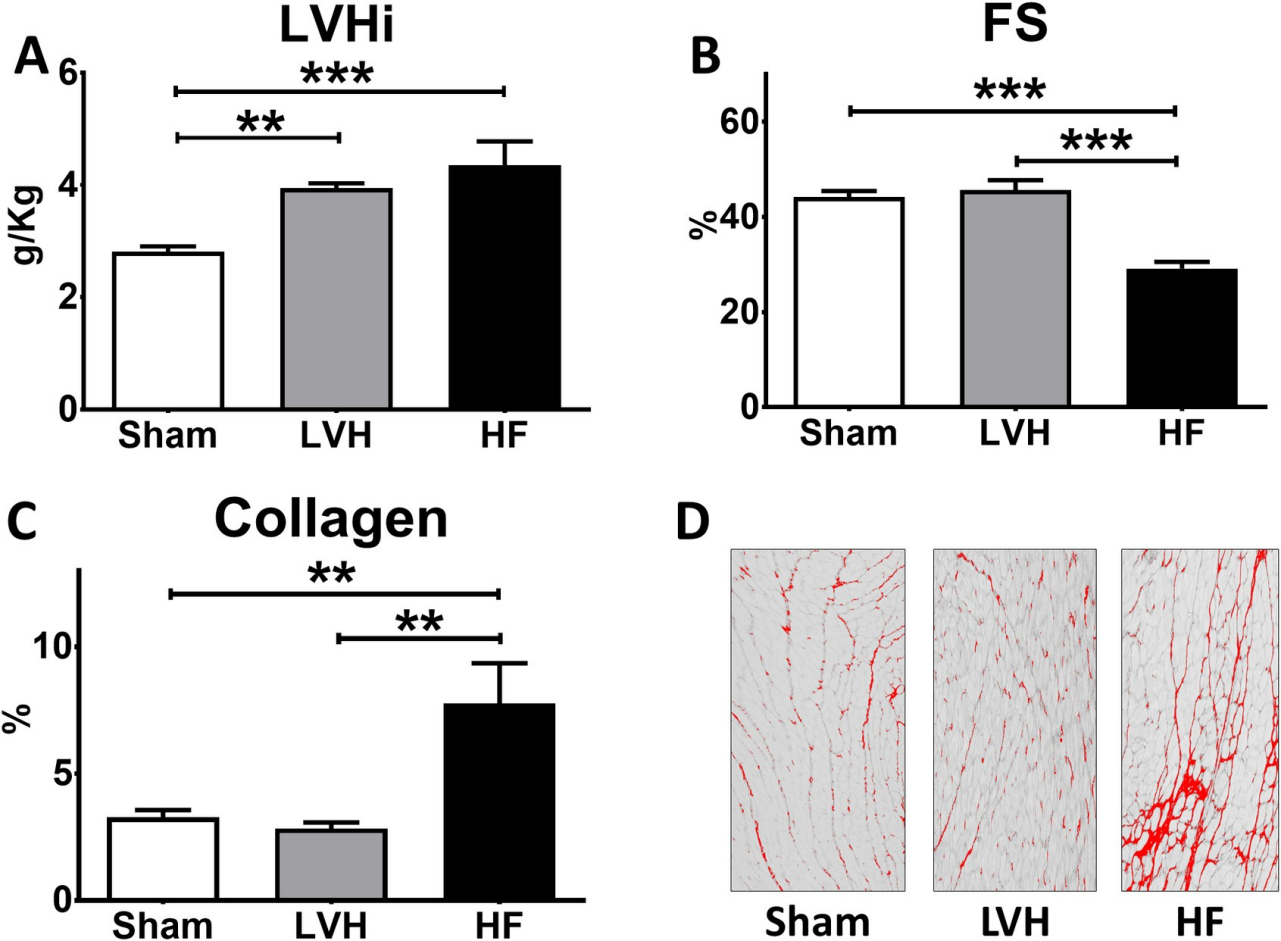
Characterization of the different cardiac remodeling stages of pressure overload. **(A)** Left ventricular mass indexed by the body weight (LVHi) in sham (Sham), left ventricular hypertrophy (LVH) and heart failure (HF) rats (ANOVA p < 0.001). **(B)** Left ventricle fractional shortening (FS) in all groups (ANOVA p < 0.001). **(C)** Collagen deposition in LV apex (ANOVA p < 0.01). **(D)** Representative images of picrosirius red staining of LV apex samples. **p < 0.01 vs Sham, ***p < 0.001 vs Sham.

From the 33 rats that underwent TAC surgery, eight (24%) presented a normal systolic function and normal cardiac mass. These rats were classified as non-successful TAC and excluded from further analyses. Fourteen rats (42%) had systolic function impairment (FS<35.2%; lowest value of sham rats) and were classified as HF rats. Eleven rats (33%) had no FS impairment but had a LVHi>3.32 g/kg (largest value of sham rats) and were classified as LVH rats. Overall, 25 rats were classified as successful TAC into two different groups: a first stage involving LV hypertrophy and normal systolic function, and a second stage characterized by FS impairment. Representative LV parasternal, M-mode echocardiographic images of all groups are shown in S2 Fig. LVHi and FS values are represented in Fig 1A and 1B.

Echocardiographic and ECG studies reflected a progressive remodeling in rats submitted to TAC. A thicker body weight-indexed IVS was found in the LVH group compared with Sham, in contrast to no differences for indexed PW (S1 Table). HF rats had a dilated LV (larger LVEDDi and LVESDi, S1 Table) compared with both Sham and LVH rats. All rats had an ECG performed before sacrifice (S2 Table). LVH and HF rats presented prolonged QT and HR-corrected QT intervals in comparison to Sham rats. No significant differences were found amongst groups for P wave, PR-interval, QRS duration and HR.

Collagen deposition in the ventricular apex was assessed with picrosirius red staining. Fibrosis was similar in LVH and Sham rats, while HF rats had a remarkable ~2.5-fold increase (Fig 1C and 1D). The magnitude of collagen deposition correlated through an apparently asymptotic curve with the degree of structural remodeling: fibrosis negatively correlated with FS and positively with LVHi, LVESDi and LVEDDi (S3A Fig).

### 3.2 Molecular validation of different stages of pressure-overload

**Fig 2:**
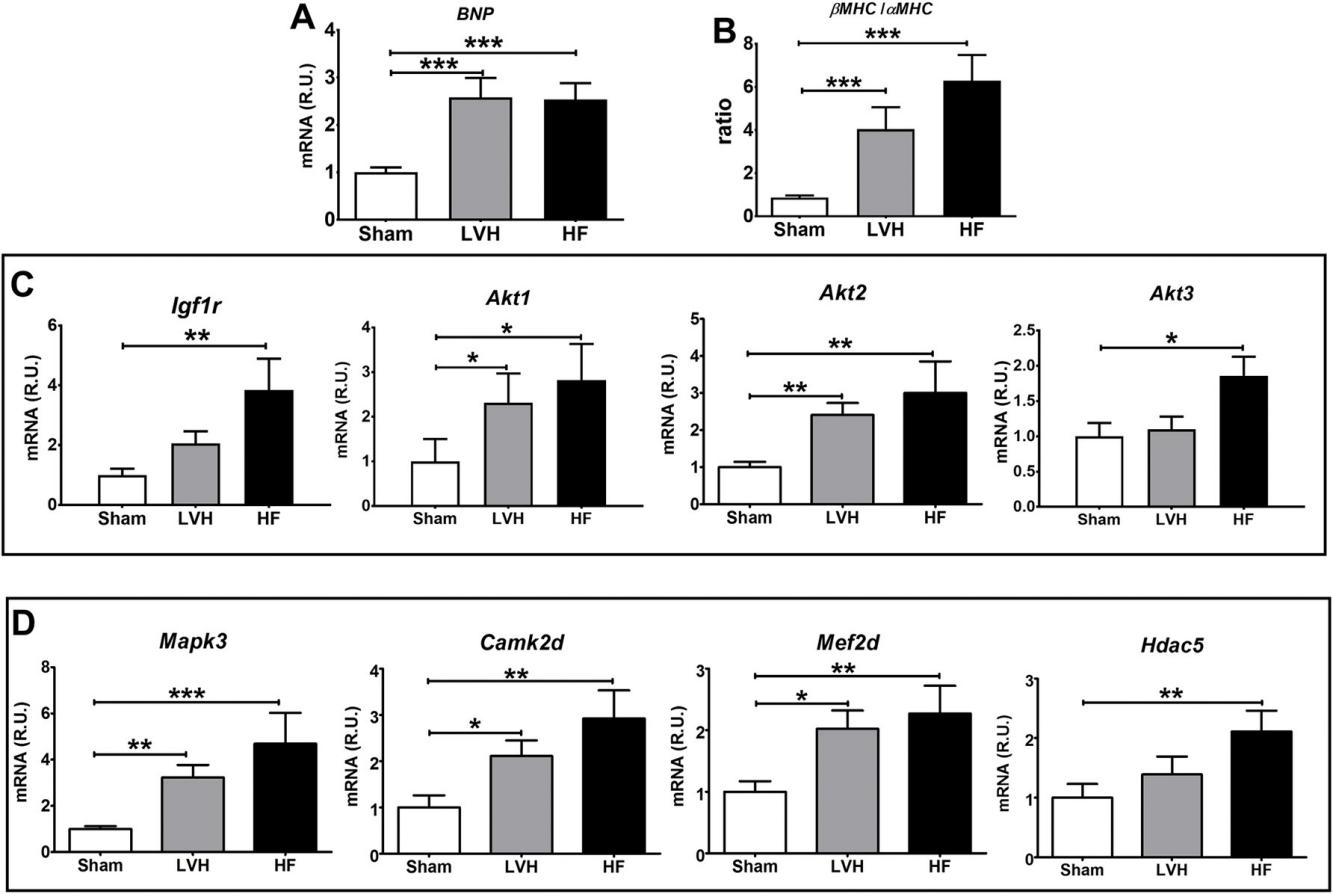
Molecular validation of different stages of LV pressure overload. **(A)** Messenger RNA levels in the LV of BNP (ANOVA p<0.001). **(B)** β myosin heavy chain (βMHC) to α myosin heavy chain (αMHC) mRNA levels ratio in the LV (ANOVA p<0.001). **(C)** Messenger RNA levels of IGF1 pathway related proteins: insulin-like growth factor 1 receptor (Igf1r) and the RAC-alpha serine/threonine-protein kinases (Akt1, Akt2 and Akt3) (ANOVA p<0.05 for all genes). **(D)** Messenger RNA levels of GPCRs pathway related proteins: mitogen-activated protein kinase 3 (Mapk3), calcium/calmodulin-dependent protein kinase type II delta subunit (Camk2d), myocyte-specific enhancer factor 2D (Mef2d) and Histone deacetylase 5 (Hdac5) (ANOVA p<0.05 for all genes). *p < 0.05 vs Sham, **p < 0.01 vs Sham, ***p < 0.001 vs Sham.

Consistent with HF progression, *BNP* mRNA levels in LV were significantly different among the three groups largely due to a 2.5-fold increase in both TAC groups (LVH and HF) compared to Sham (ANOVA, p<0.001, Fig 2A). The *βMHC/αMHC* ratio also differed with increasing expression from Sham to LVH rats (4-fold) to HF rats (6-fold increase in comparison with Sham) (ANOVA, p<0.001, Fig 2B).

Analysis of cardiac hypertrophy related genes revealed a characteristic, progressive hypertrophic remodeling pattern from Sham to LVH to HF rats. The complete report of results for all genes is provided in S3 Table. Increased LV mRNA levels of proteins involved in the IGF1 (Insulin-like growth factor 1, Fig 2C) pathway such as IGF1 receptor (p<0.05) and the *Akt* (RAC-alpha serine/threonine-protein kinase) kinases (*Akt1*, p<0.05 and *Akt2*, p<0.01) were found in LVH and HF rats compared to Sham, while *Akt3* did only increased in HF rats (p<0.05). We also found an apparent activation of downstream G protein-coupled receptors (Fig 2D and S3 Table) pathways such as *Mapk3* (Mitogen-activated protein kinase 3, p<0.01), *Camk2d* and *Camk2g* (Calcium/calmodulin-dependent protein kinase type II subunits delta and gamma, p<0.01 in both cases), *Mef2c* and *Mef2d* (Myocyte-specific enhancer factor 2C and 2D, p<0.01 in both cases) and *Hdac5* (Histone deacetylase 5, p<0.05).

Left ventricular *BNP* mRNA expression correlated with all the echocardiographic measures except for FS and LVEF (Table 1).

**Table 1:** Correlation of BNP and Axl mRNA levels in LV with ecocardiographic parameters.

	LV-*BNP* (log)		LV-*Axl* (log)	
	R	p	R	p
**LVHi (g/Kg)**	0.62	<0.001	0.42	0.01
**IVSi (mm/Kg)**	0.61	<0.001	0.23	0.22
**PWi (mm/Kg)**	0.47	0.006	-0.13	0.48
**LVEDDi (mm/Kg)**	0.42	0.02	-0.06	0.76
**LVESDi (mm/Kg)**	0.41	0.02	0.07	0.71
**FS (%)**	-0.31	0.08	-0.28	0.13
**LVEF (%)**	-0.33	0.06	-0.27	0.12
**LADi (mm/Kg)**	0.56	0.04	-0.13	0.66

LV-BNP (log): log-transformed mRNA levels in the LV; LV-Axl (log) log-transformed mRNA levels in the LV; LVHi: left ventricular hypertrophy index; IVSi: body weight (BW)-indexed interventricular septum thickness (IVSi); PWi BW-indexed posterior wall indexed; LVEDDi BW-indexe left ventricle end-diastolic diameter; LVESDi: BW-indexed left ventricle end-systolic diameter; FS: fractional shortening; LVEF: left ventricle ejection fraction; LADi: BW-indexed left atrial diameter.

Fibrotic and extracellular remodeling markers *Tgfβ1*, *Mmp2*, *Timp1*, *Col1a1*, *Col3a1*, fibronectin and vimentin demonstrated a complex balance underlying myocardial fibrosis at different overload stages. Expression of the profibrotic *Tgfβ1* cytokine and the extracellular matrix remodeling factors *Mmp2* and *Timp1* were significantly increased in both LVH and HF groups compared to Sham (S3B Fig). *Col1a1* and *Col3a1* mRNA levels were also different between groups (ANOVA, p<0.01 and p<0.05, respectively), with higher values in LVH rats than in Sham rats (S3C Fig). Differences between groups were also found with the profibrotic factors fibronectin and vimentin (S3C Fig) with higher values in both LVH and HF than in Sham rats.

### 3.3 *Axl* expression is increased in LV in TAC animals

**Fig 3:**
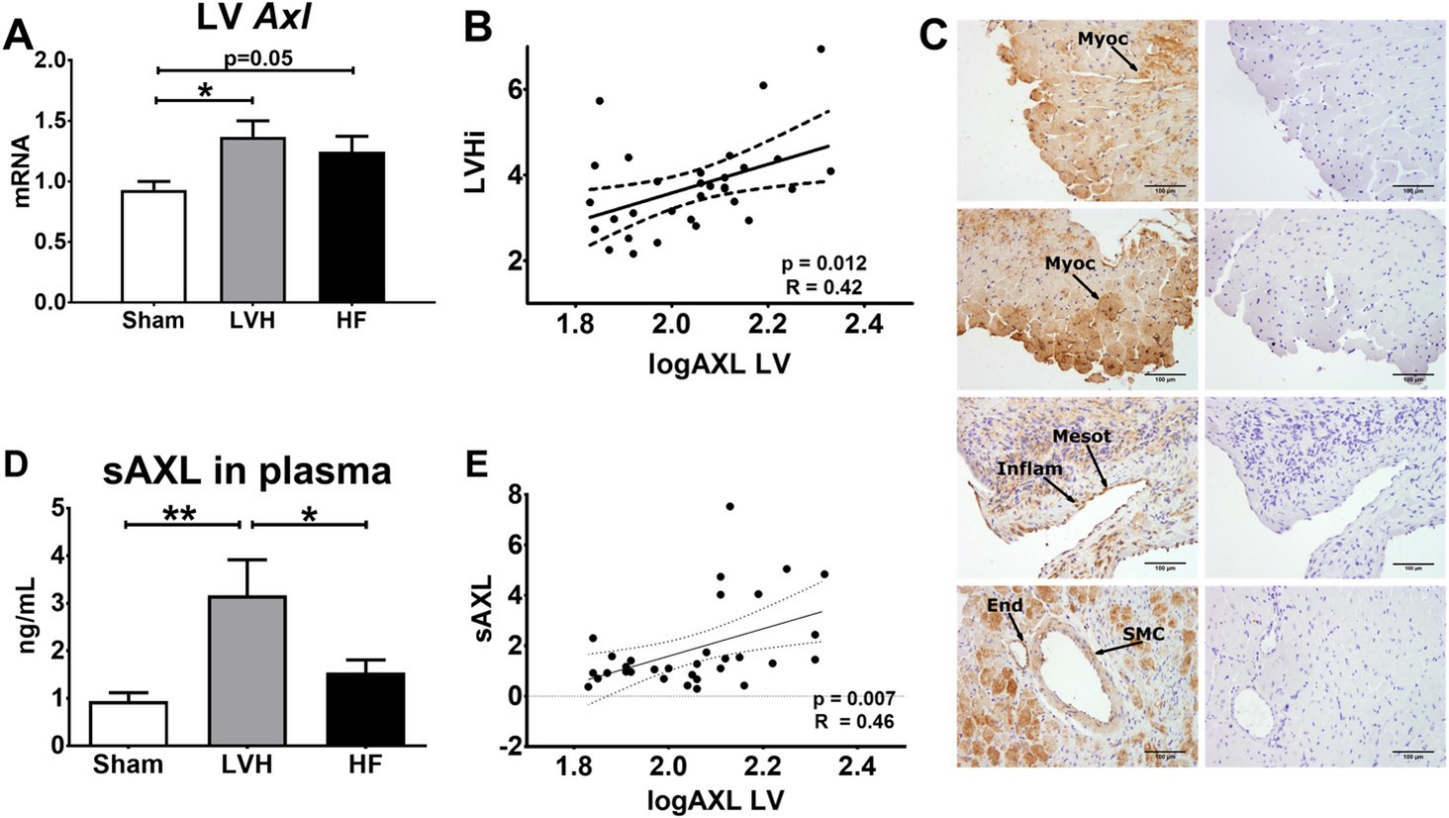
Axl expression in the LV at different stages of TAC. **(A)** Axl left ventricle (LV) mRNA levels in all groups (ANOVA p<0.05). **(B)** Linear correlation between LV logAxl mRNA levels and the LV hypertrophy index (LVHi). **(C)** Representative AXL immunohistochemistry microphotographs from the LV of LVH (four upper pannels) and HF (four lower pannels) rats. In right panels, myocytes (Myoc), inflammatory cells (Inflam), mesothelial cells (Mesot), endothelial cells (End) and smooth muscle cells (SMC) staining are indicated with black arrows. Negative staining controls are presented in left panels. **(D)** sAXL levels in plasma in all groups (ANOVA p<0.01). **(E)** Linear correlation between logAxl mRNA levels in LV and sAxl in plasma. *p < 0.05 vs Sham, **p < 0.01 vs Sham.

**Fig 4:**
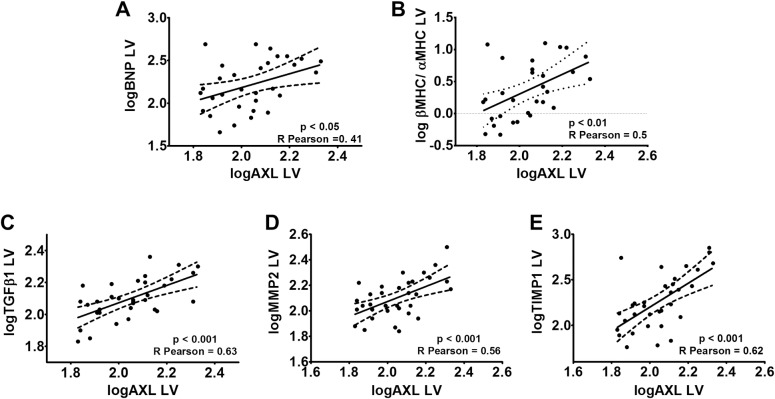
Axl mRNA levels in LV correlate with remodeling factors. Linear correlation between logAxl mRNA levels and logBNP mRNA levels in LV **(A)**, the logRatio of β myosin heavy chain (βMHC) over α myosin heavy chain (αMHC) mRNA levels in LV **(B)**, the logTgfβ1 mRNA levels in LV **(C)**, the logMmp2 mRNA levels in LV **(D)**, the logTimp1 mRNA levels in LV **(E)**.

Left ventricular *Axl* mRNA levels significantly differed among the three groups (Fig 3A). *Axl* expression was higher in LVH and HF rats compared with Sham animals; expression was similar in LVH and HF animals. A similar pattern was found for AXL protein levels in Western-blot experiments, although this did not reach statistical significance (p = 0.067 for Sham vs LVH) likely due to the lower sample size (S4 Fig). *Axl* mRNA levels positively correlated with a deeper structural echocardiographic remodeling as assessed with LVHi (Fig 3B), but not with systolic function (Table 1). At the molecular level, *Axl* mRNA levels showed a moderate, positive correlation with *BNP* (Fig 4A), *βMHC/αMHC* ratio (Fig 4B), *Tgfβ*1 (Fig 4C), *Mmp2* (Fig 4D) and *Timp1* (Fig 4E) mRNA levels. In contrast, no correlation was found between *Axl* and *Col1a1* (p = 0.058, R Pearson = 0.33), *Col3a1* (p = 0.06, R Pearson = 0.33), fibronectin (p = 0.47, R Pearson = 0.13) and vimentin (p = 0.10, R Pearson = 0.29) mRNA levels, and the apex collagen percentage (p = 0.13, R Pearson = 0.35)

AXL protein expression in the LV localized in myocytes, inflammatory and mesothelial cells, as well as in vascular endothelial and smooth muscle cells (Fig 3C).

Plasma concentration of soluble Axl was higher in LVH compared with Sham and HF rats (Fig 3D). Soluble AXL yielded a moderate to good estimate of LV *Axl* mRNA levels (Fig 3E).

**Fig 5:**
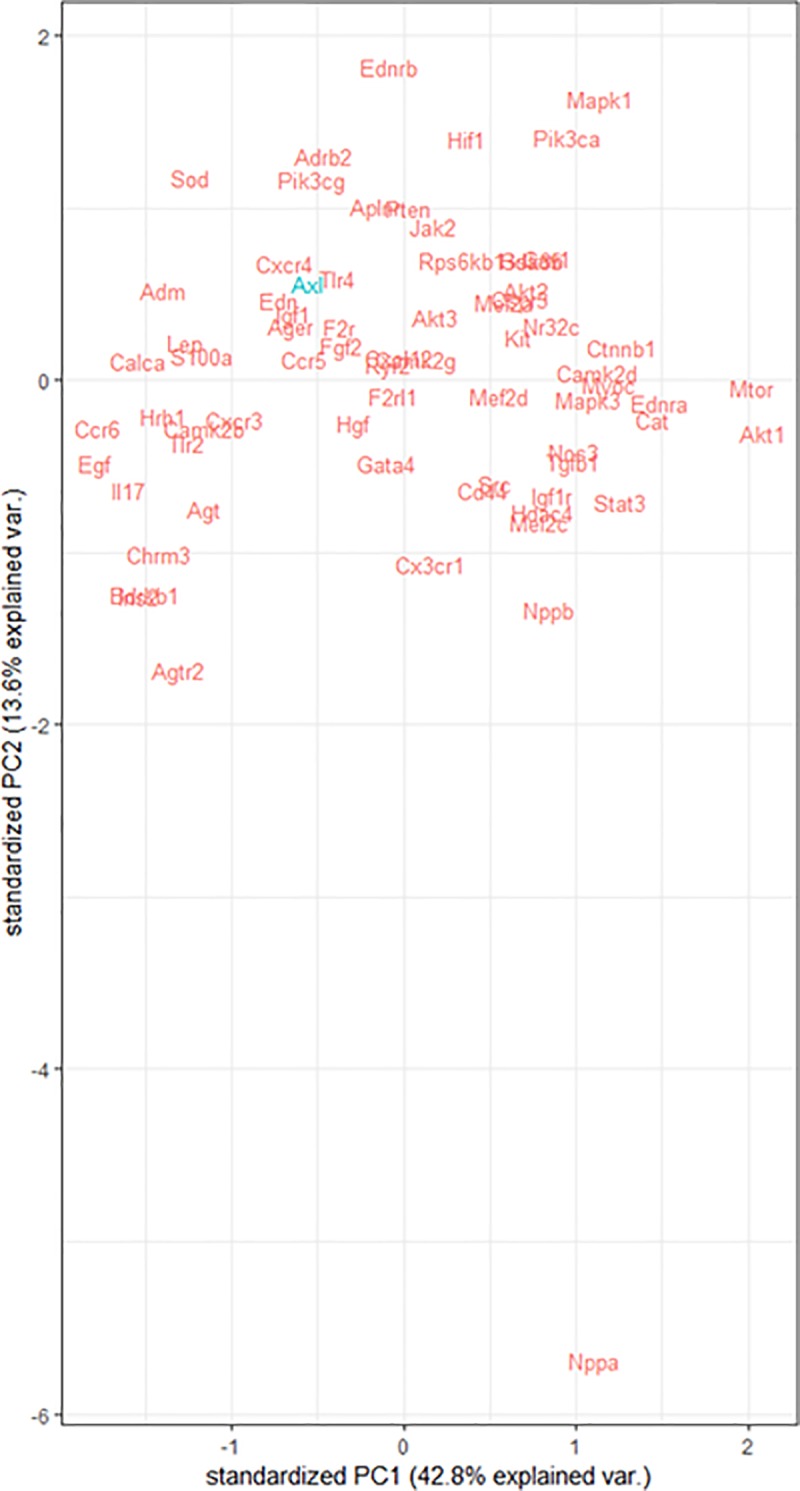
Correlation of Axl mRNA expression to hypertrophy mediators. The 2 main principal components in a PCA analysis involving all tested genes are plotted. Axl (in blue) and cardiac remodeling factors (in red) mRNA levels.

In view of the association between AXL and cardiac hypertrophy, we aimed at further exploring *Axl* co-expression patterns with hypertrophic factors. *Axl* mRNA levels were analyzed in the context of hypertrophy mediators. A joint principal component analysis showed that *Axl* expression clustered with other well-known hypertrophy factors such as *Edn* (Endothelin-1), *Tlr4* (Toll-like receptor 4 precursor), *Cxcr4* (C-X-C chemokine receptor type 4) and *Igf1* (Insulin-like growth factor I) (Fig 5).

### 3.4 *Axl* expression is not increased in LK or aAo in TAC animals

**Fig 6:**
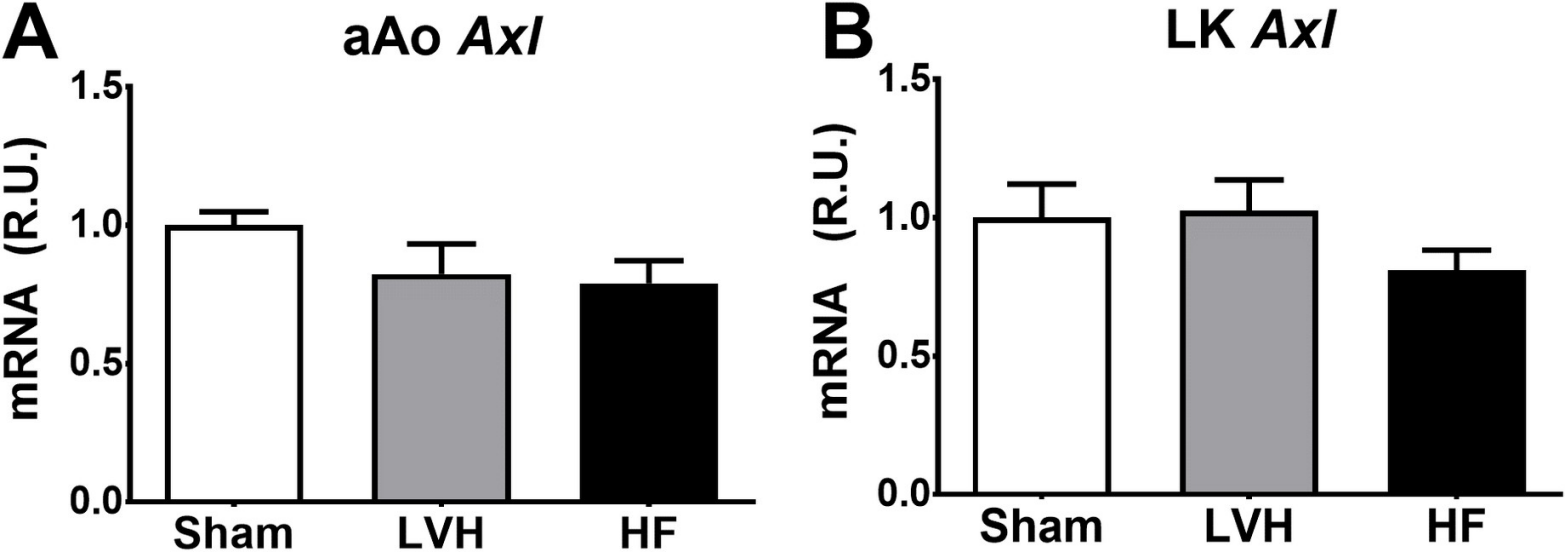
Axl expression in LK and aAo in the TAC model progression to HF. Ascending aorta (aAo, panel **A**) and left kidney (LK, panel **B**) Axl mRNA levels in sham, left ventricular hypertrophy (LVH) and heart failure rats (HF).

To test whether HF prompted remote changes in *Axl* expression, expression in the LK and aAo was quantified. We found no differences between Sham, LVH and HF rats in either LK or aAo (Fig 6A and 6B).

## 4. Discussion

This work describes for the first time changes in the LV expression of the tyrosine kinase receptor *Axl* in a pressure-overload murine model. Our main findings are that (a) *Axl* mRNA levels are increased in the LV of rats subjected to pressure-overload, (b) *Axl* expression correlates with the degree of LV hypertrophy but not with systolic function impairment, (c) circulating sAXL levels estimate LV expression and are higher in plasma from LVH rats compared to Sham and HF rats, and (d) renal and vascular *Axl* expression are not altered in this model.

### 4.1 AXL participates in pressure overload cardiac remodeling

The role of *Axl* in heart disease is still under debate. In transgenic mice selectively overexpressing cardiac TNF-*α*, the expression of TAM family members *Tyro3* and *MerTK* was up-regulated in end-stage HF animals, while *Axl* and *Gas6* remained unaltered; unfortunately, no time-course experiments at earlier time points were performed [26]. Conversely, mice lacking *Gas6*, the only known Axl ligand, were protected from pressure-overload and developed a blunted hypertrophic response and dysfunction in comparison with wild-type animals [17]. In a different setting, Zhang et al. showed that Gas6/Axl may have a protective role in a rat model of myocardial ischemia/reperfusion injury [16].

In order to better characterize the role of AXL in HF and understand its role as a biomarker, we studied *Axl* expression in rats subjected to TAC. Transverse aortic constriction is a well-established model that mimics cardiac remodeling occurring after the instauration of LV pressure-overload where different degrees of constriction trigger uneven remodeling levels. Thereby, this model replicates the timeline stages that lead to the initial LVH and, eventually, HF [22,27]. In the LV of TAC rats, *Axl* mRNA expression was increased even in the absence of overt HF, and correlated with both *BNP* expression and the *βMHC/αMHC* ratio; the later are standard measures of pressure-overload cardiac remodeling and dysfunction [17,27]. Nevertheless, differences between these are to be considered. *BNP* showed a better correlation with systolic function than *Axl*, in keeping with recent clinical data [20]. The switch from a predominantly alpha-MHC isoform to a predominantly beta-MHC isoform progresses from Sham to LVH to HF rats [27], while *Axl* mRNA expression was similar in both TAC groups.

#### 4.1.1 Axl and cardiac hypertrophy

In the LV of this pressure-overload model, we found characteristic hypertrophy signatures in LVH and HF rats. Specifically, we found a progressive (Sham to LVH to HF) activation of IGF1 and GPCRs downstream pathways (Fig 2), including an increased expression of both endothelin receptors A and B (S3 Table). *Axl* mRNA expression in LV correlated with the LVHi and with well-established hypertrophy factors such as *Edn* (Endothelin-1), *Igf1* (Insulin-like growth factor I), *Tlr4* (Toll-like receptor 4 precursor) and *Cxcr4* (C-X-C chemokine receptor type 4). In plasma, sAXL was increased only in LVH rats but not in HF rats. All these results are concordant with a role of *Axl* in the early hypertrophic response of pressure-overload triggered cardiac remodeling, possibly substantiating the lack of changes in late HF stages in previous works [26].

#### 4.1.2 Axl and myocardial fibrosis

The *Axl* role in cardiac fibrosis is not conclusive from our results as it is in liver [4,5] or lung [9] fibrosis. A correlation between *Axl* and *Tgfβ1*, *Mmp2* and *Timp1* expression suggests that *Axl* has a role in early cardiac changes, but we did not find correlation with *Col1a1*, *Col3a1*, fibronectin and vimentin mRNA levels in LV, nor with the histological fibrosis. Tgf*β*1 is a key factor in pathological fibrosis and a tight relationship of *Axl* with Tgf*β*1 can be implied from the reduced expression of *Tgfβ1* in *Axl* knockdown [8].

The progression of heart fibrosis after acute myocardial infarction (AMI) in an early and a late phase parallels our results. In the early phase, Tgf*β*1 stimulates fibroblast chemotaxis, proliferation and transformation to myofibroblast and MMPs and TIMPs expression increases. On the contrary, collagen-I and III accumulation occurs in late fibrosis when there is replacement of myocytes and scar formation [28]. Altogether, it cannot be discarded that Axl triggers Tgf*β*1 expression in the early phase and consequently the whole fibrotic cascade.

### 4.2 Soluble AXL as a plasmatic biomarker

Soluble AXL has been used as a plasmatic prognostic biomarker in several heart diseases and settings. In cardiac transplant recipients, sAXL values were higher in those presenting with adverse CV events, an association that remained significant after multivariate adjustment [18]. After an ST-elevation AMI, higher sAXL levels flag those patients who will develop HF and an adverse LV remodeling during follow-up [19]. We have reported that sAXL is a good prognostic marker of the HF major events all-cause mortality, transplantation and HF hospitalizations at 1 year [20] and at 3.6 years follow-up [29]. The higher sAXL levels in plasma from LVH rats, along previous clinical data [18–20,29], substantiate the use of sAXL as a plasmatic biomarker in HF patients. In contrast with our observations that *Axl* may play a role in early remodeling, sAXL was higher in patients with NYHA functional class III than II [20]. Nevertheless, functional class assessment is determined by several factors beyond, and may not fully be explained by, cardiac remodeling. Also, the heterogeneity within HF patients regarding etiology and evolution time could account for the clinic-experimental differences encountered.

#### 4.2.1 Circulating AXL may be of cardiac origin in HF patients

*Axl* has been involved in remodeling processes in vessel walls and in the kidney, among others. It is therefore possible that, in HF patients, sAXL does not reflect cardiac remodeling, but rather the co-existence of other comorbidities or the systemic impact of HF. We therefore quantified *Axl* expression in the kidney and the ascending aorta wall. On one hand, *Axl* has been shown to contribute to the pathology of several glomerulonephritis and kidney conditions [10,11,30], but our results show that *Axl* expression is not altered in the kidney. On the other hand, *Axl* has been demonstrated to mediate VSMC migration and proliferation in vessel walls after vascular injury [31], hypertension [32] and flow-induced vascular injury [15,31]. Due to the increased wall stretch in the proximal side of the aortic constriction, we quantified *Axl* mRNA levels in aAo, where we found a similar expression of *Axl* in Sham, LVH and HF rats.

In contrast, we found AXL synthesis in LV myocytes, amongst other cellular types. Moreover, there was a good correlation between cardiac *Axl* mRNA levels and peripheral sAXL protein. Altogether, these data support that sAXL originates from the LV of HF patients. Nevertheless, we cannot exclude that with worsening of kidney function in later HF stages, an sAXL increase could partially arise from a lack of renal excretion.

### 4.3 Limitations

Some limitations of our work should be acknowledged. First, data from animal models, in which acute pressure overload is imposed to a previously healthy LV might not be comparable to chronic cardiac conditions in humans. Second, up to 24% of rats submitted to TAC did not develop signs of LVH nor of HF. In this regard, a report with mice followed with cardiac magnetic resonance confirmed the high variability of response to TAC; band internalization could account for a lack of cardiac changes in some animals [33].

## 5. Conclusions

*Axl* expression is increased in the LV of compensated and decompensated forms of pressure-overload insults, while its expression in the kidney and ascending aorta remains unaltered. The higher sAXL plasma levels in LVH rats and the relationship between *Axl* mRNA and LVHi suggest a role of *Axl* in early cardiac remodeling stages. Nevertheless, further research will be needed to gain more insight about the *Axl* role in the cardiac remodeling process that leads to HF.

## Supporting information

S1 Fig Echocardiographic parameters in sham rats. **(A)** Frequency distribution of fractional shortening (FS) from sham rats represented as an histogram, n (number of rats), the arrow points to the LLN (lower limit of normality). **(B)** Frequency distribution of left ventricular hypertrophy index (LVHi) from sham rats represented as an histogram, the arrow points to the ULN (upper limit of normality).
(TIF)

S2 Fig Representative echocardiographic recordings in all groups. Recordings obtained in the M-mode in a parasternal longitudinal view of the LV.
(TIF)

S3 Fig Characterization of the TAC model progression to HF. **(A)** Correlation between collagen in apex and left ventricle fractional shortening (FS), left ventricular mass index (LVHi), body weight (BW)-indexed left ventricular end-systolic diameter (LVESDi), and BW-indexed left ventricular end-diastolic diameter (LVEDDi). **(B)** Messenger RNA levels in the LV of *Tgfβ1* (ANOVA p<0.001), *Mmp2* (ANOVA p<0.01), and *Timp1* (ANOVA p<0.001). **(C)** Messenger RNA levels in the LV of *Col1a* (ANOVA, p<0.05), *Col3a* (ANOVA p<0.05), *Fibron* (ANOVA p<0.01), and vimentin (ANOVA p<0.05). *p < 0.05 vs Sham, **p < 0.01 vs Sham, ***p<0.001 vs Sham.
(TIF)

S4 Fig AXL protein levels in the LV. (TIF)

S1 Table Echocardiographic data in all groups. LVH: left ventricular hypertrophy rats; HF: heart failure rats; LVHi: left ventricular mass indexed by rat body weight (BW) at the sacrifice; IVSi: BW-indexed interventricular septum thickness; PWi: BW-indexed posterior wall thickness; LVEDDi: BW-indexed left ventricular end-diastolic diameter (LVEDDi); LVESDi: BW-indexed left ventricular end-systolic diameter; FS: fractional shortening; LVEF: left ventricle ejection fraction; LADi: BW-indexed left atrial diameter indexed. **p<0.01 vs Sham, ***p<0.001 vs Sham, ^§§^p<0.01 vs LVH, ^§§§^p<0.001 vs LVH.
(DOCX)

S2 Table ECG parameters in all groups. LVH: left ventricular hypertrophy rats; HF: heart failure rats; bpm: beats per minute (bpm). **p<0.01 vs Sham.
(DOCX)

S3 Table mRNAs analyzed with the bio-rad rat cardiac hypertrophy plate. Data are provided with the 2^-ΔΔCt^ method, where fold change in left ventricular hypertrophy rats (LVH) and heart failure rats (HF) are compared to Sham. Whenever amplification was detected in <70% of samples, non-applicable (NA) is reported. *p<0.05 vs Sham, **p<0.01 vs Sham, ***p<0.001 vs Sham.
(DOCX)

S1 Methods RNA isolation and real time assays. (DOCX)

S2 Methods AXL protein levels quantification in plasma and cardiac tissue. (DOCX)

S3 Methods AXL immunohistochemistry. (DOCX)
