# Supplementary figures and images for: Axl expression is increased in early stages of left ventricular remodeling in an animal model with pressure-overload
Source: PLoS One. 2019 Jun 10;14(6):e0217926. doi: 10.1371/journal.pone.0217926 (PMC6557565; doi:10.1371/journal.pone.0217926)

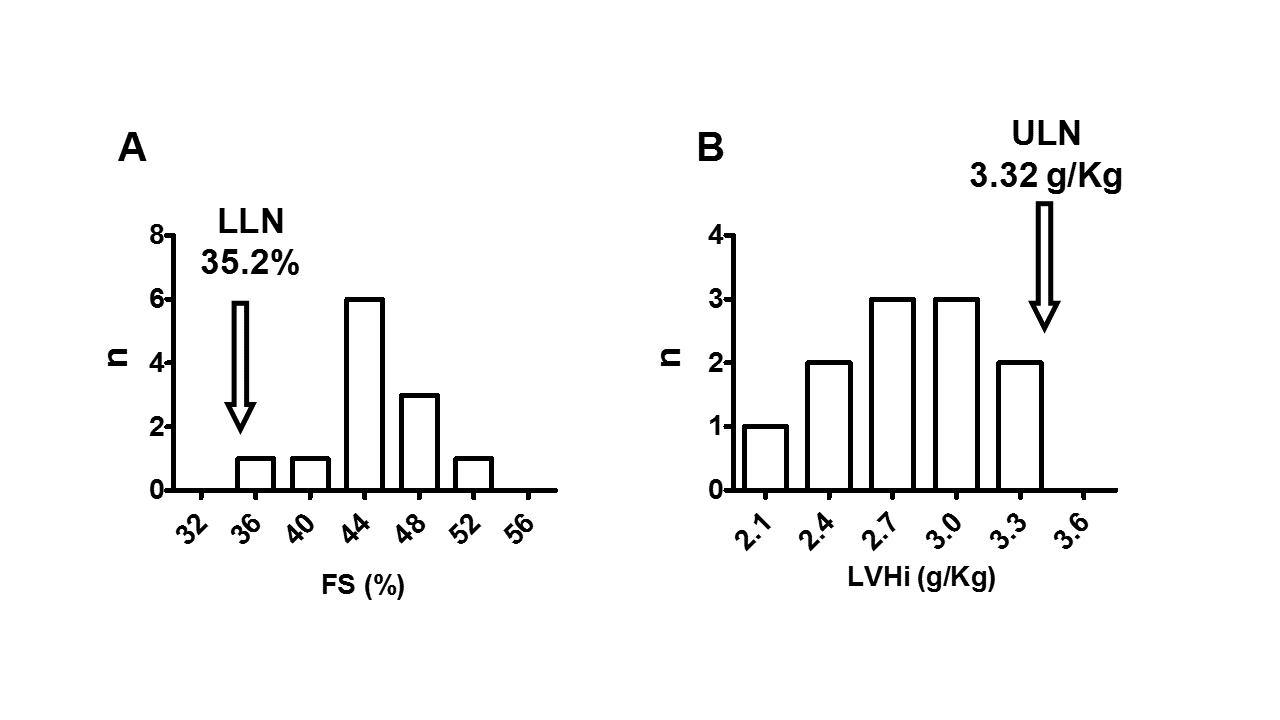

Supplement: S1 Fig — (A) Frequency distribution of fractional shortening (FS) from sham rats represented as an histogram, n (number of rats), the arrow points to the LLN (lower limit of normality). (B) Frequency distribution of left ventricular hypertrophy index (LVHi) from sham rats represented as an histogram, the arrow points to the ULN (upper limit of normality). (TIF) [file pone.0217926.s001.tif]

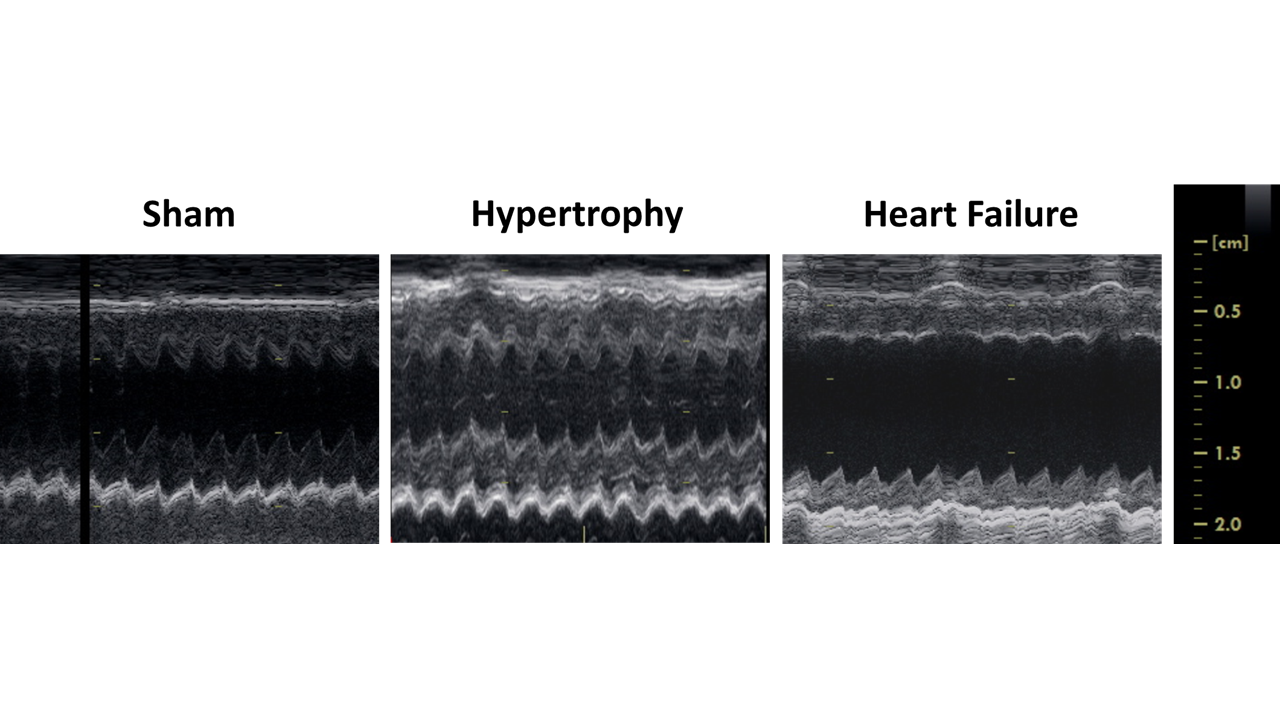

Supplement: S2 Fig — Recordings obtained in the M-mode in a parasternal longitudinal view of the LV. (TIF) [file pone.0217926.s002.tif]

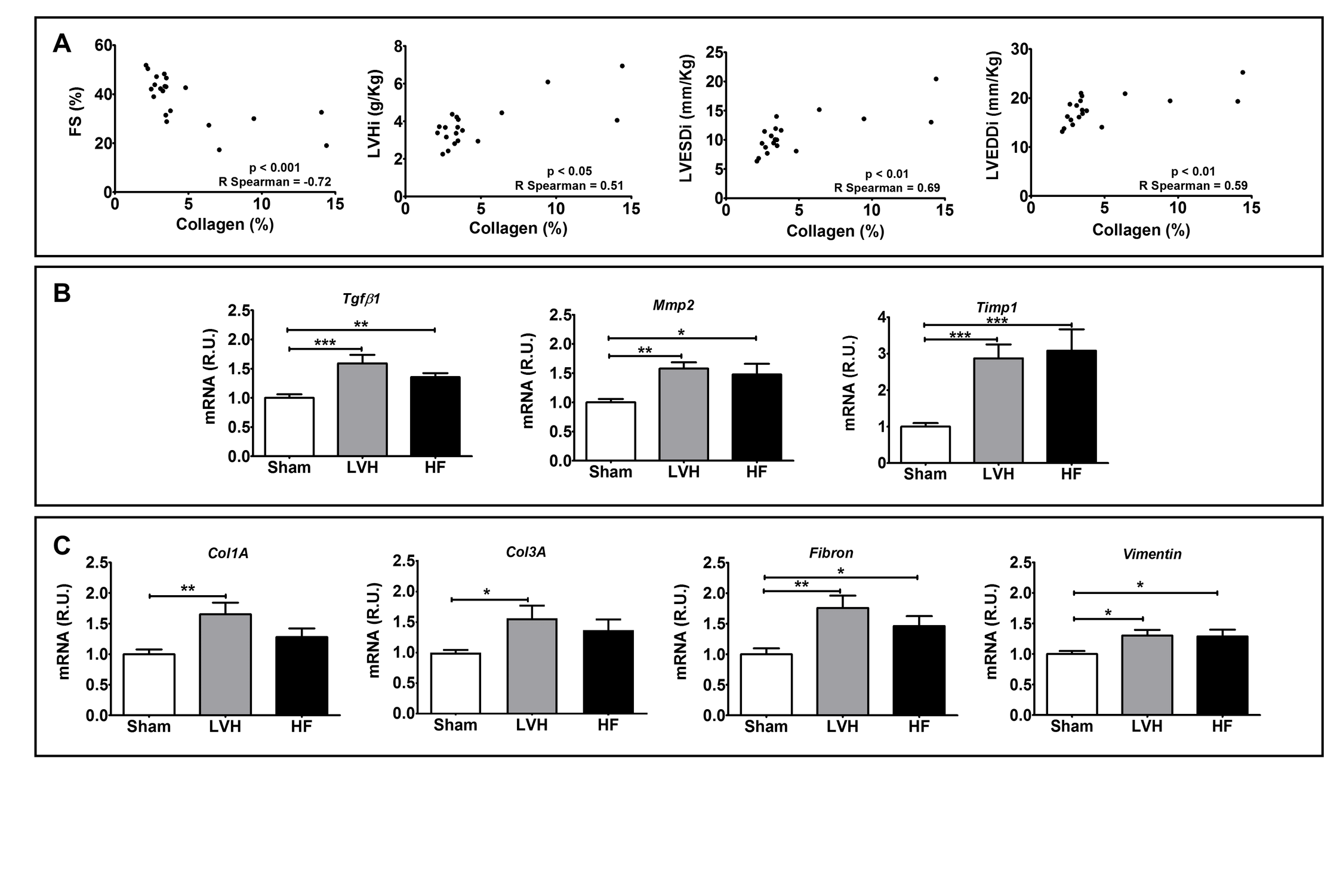

Supplement: S3 Fig — (A) Correlation between collagen in apex and left ventricle fractional shortening (FS), left ventricular mass index (LVHi), body weight (BW)-indexed left ventricular end-systolic diameter (LVESDi), and BW-indexed left ventricular end-diastolic diameter (LVEDDi). (B) Messenger RNA levels in the LV of Tgfβ1 (ANOVA p<0.001), Mmp2 (ANOVA p<0.01), and Timp1 (ANOVA p<0.001). (C) Messenger RNA levels in the LV of Col1a (ANOVA, p<0.05), Col3a (ANOVA p<0.05), Fibron (ANOVA p<0.01), and vimentin (ANOVA p<0.05). *p < 0.05 vs Sham, **p < 0.01 vs Sham, ***p<0.001 vs Sham. (TIF) [file pone.0217926.s003.tif]

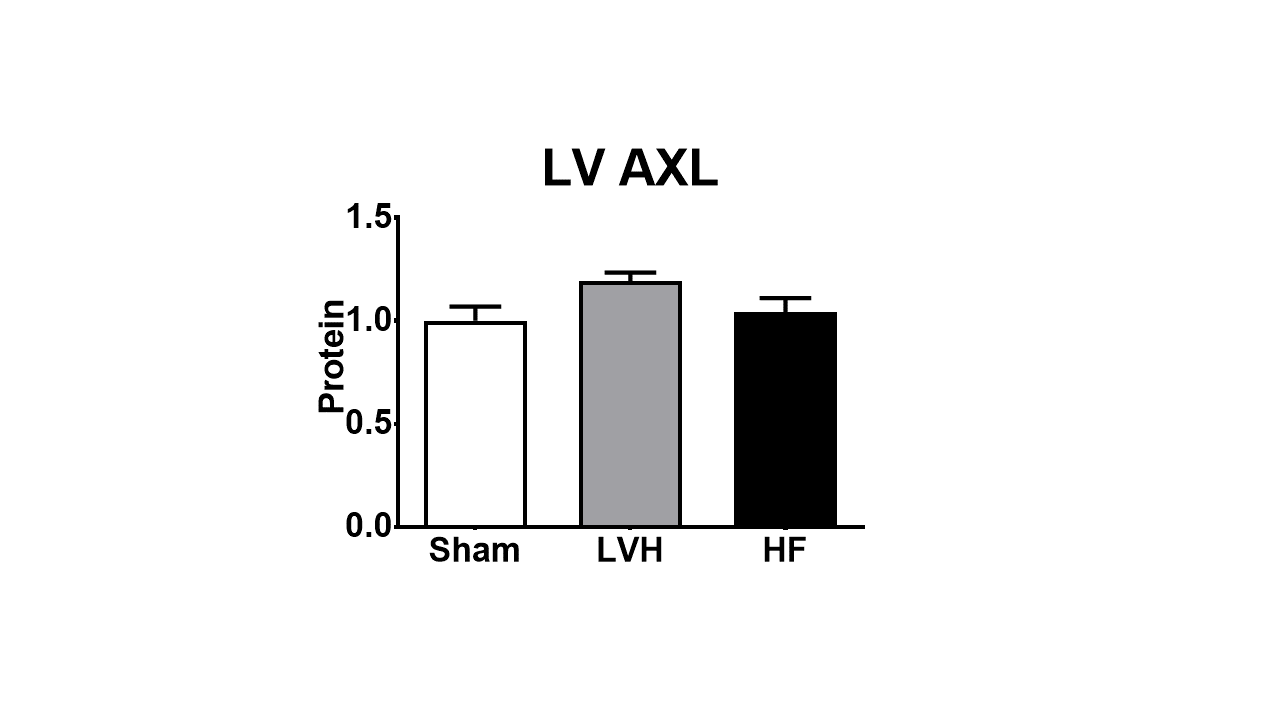

Supplement: S4 Fig — (TIF) [file pone.0217926.s004.tif]
