# Supplementary material for: Axl expression is increased in early stages of left ventricular remodeling in an animal model with pressure-overload
Source: PLoS One. 2019 Jun 10;14(6):e0217926. doi: 10.1371/journal.pone.0217926 (PMC6557565; doi:10.1371/journal.pone.0217926)
Supplement: S1 Table — LVH: left ventricular hypertrophy rats; HF: heart failure rats; LVHi: left ventricular mass indexed by rat body weight (BW) at the sacrifice; IVSi: BW-indexed interventricular septum thickness; PWi: BW-indexed posterior wall thickness; LVEDDi: BW-indexed left ventricular end-diastolic diameter (LVEDDi); LVESDi: BW-indexed left ventricular end-systolic diameter; FS: fractional shortening; LVEF: left ventricle ejection fraction; LADi: BW-indexed left atrial diameter indexed. **p<0.01 vs Sham, ***p<0.001 vs Sham, §§p<0.01 vs LVH, §§§p<0.001 vs LVH. (DOCX) [file pone.0217926.s005.docx]

|  | ***Sham***  ***(n=12)*** | ***LVH***  ***(n=11)*** | ***HF***  ***(n=14)*** | ***ANOVA p*** |
| --- | --- | --- | --- | --- |
| ***LVHi (g/Kg)*** | 2.8 ± 0.1 | 3.9 ± 0.1** | 4.4±0.4*** | <0.001 |
| ***IVSi (mm/Kg)*** | 4.74 ± 0.2 | 6.01 ± 0.32* | 5.63±0.41* | 0.03 |
| ***PWi (mm/Kg)*** | 5.38 ± 0.26 | 5.93 ± 0.27 | 6.01±0.37 | 0.3 |
| ***LVEDDi (mm/Kg)*** | 16.72 ± 0.54 | 17.01 ± 0.69 | 20.1±1**^/§§^ | 0.007 |
| ***LVESDi (mm/Kg)*** | 9.32 ± 0.43 | 9.27 ± 0.56 | 14.15±0.84***^/§§§^ | <0.001 |
| ***FS (%)*** | 44.2 ± 1.3 | 70.1 ± 2.1 | 29.2±1.4***^/§§§^ | <0.001 |
| ***LVEF (%)*** | 68 ± 1 | 70 ± 25 | 50±2***^/§§§^ | <0.001 |
| ***LADi (mm/Kg)*** | 11.70 ± 0.35 | 12.26 ± 0.31 | 12.65±0.98 | 0.62 |
