# Supplementary material for: Axl expression is increased in early stages of left ventricular remodeling in an animal model with pressure-overload
Source: PLoS One. 2019 Jun 10;14(6):e0217926. doi: 10.1371/journal.pone.0217926 (PMC6557565; doi:10.1371/journal.pone.0217926)
Supplement: S2 Table — LVH: left ventricular hypertrophy rats; HF: heart failure rats; bpm: beats per minute (bpm). **p<0.01 vs Sham. (DOCX) [file pone.0217926.s006.docx]

|  | ***Sham***  ***(n=12)*** | ***LVH***  ***(n=11)*** | ***HF***  ***(n=14)*** | ***ANOVA p*** |
| --- | --- | --- | --- | --- |
| ***Heart rate (bpm)*** | 388±9 | 357±9 | 376±11 | 0.14 |
| ***PR interval (ms)*** | 49.3±1.5 | 51.7±1.3 | 50.5±2.1 | 0.69 |
| ***QRS duration (ms)*** | 18.5±0.6 | 19.1±0.5 | 19.8±1 | 0.47 |
| ***QT interval (ms)*** | 73.9±6 | 97.7±4** | 96.9±4.2** | 0.004 |
| ***QTc interval (ms)*** | 59.3±4.8 | 75.1±2.5** | 75.7±2.4** | 0.005 |
| ***P duration (ms)*** | 16.3±0.6 | 17.4±1.6 | 17.1±1.3 | 0.79 |
| ***P amplitude (µV)*** | 92±4 | 106±9 | 104±10 | 0.46 |
| ***R amplitude (µV)*** | 596±89 | 703±74 | 568±52 | 0.49 |
| ***T amplitude (µV)*** | 75±9 | 91±13 | 102±12 | 0.19 |
