# Supplementary material for: Axl expression is increased in early stages of left ventricular remodeling in an animal model with pressure-overload
Source: PLoS One. 2019 Jun 10;14(6):e0217926. doi: 10.1371/journal.pone.0217926 (PMC6557565; doi:10.1371/journal.pone.0217926)
Supplement: S3 Table — Data are provided with the 2-ΔΔCt method, where fold change in left ventricular hypertrophy rats (LVH) and heart failure rats (HF) are compared to Sham. Whenever amplification was detected in <70% of samples, non-applicable (NA) is reported. *p<0.05 vs Sham, **p<0.01 vs Sham, ***p<0.001 vs Sham. (DOCX) [file pone.0217926.s007.docx]

|  | ***Sham*** | ***LVH*** | ***HF*** | *ANOVA, p* |
| --- | --- | --- | --- | --- |
| *Ace3* | NA | | |  |
| *Adm* | 1 ± 0.17 | 0.88 ± 0.4 | 0.71 ± 0.17 | 0.39 |
| *Adrb2* | 1 ± 0.17 | 0.7 ± 0.13 | 0.95 ± 0.09 | 0.17 |
| *Ager* | 1 ± 0.14 | 1.26 ± 0.29 | 1.54 ± 0.33 | 0.65 |
| *Agt* | 1 ± 0.2 | 1.62 ± 0.5 | 1.21 ± 0.34 | 0.69 |
| *Agtr1a* | 1 ± 0.2 | 0.73 ± 0.14 | 1.03 ± 0.21 | 0.88 |
| *Agtr2* | 1 ± 0.35 | 1.65 ± 0.43 | 0.9 ± 0.31 | 0.3 |
| *Akt1* | 1 ± 0.5 | 2.31 ± 0.66* | 2.82 ± 0.81* | 0.02 |
| *Akt2* | 1 ± 0.14 | 2.41 ± 0.32** | 3 ± 0.85** | 0.006 |
| *Akt3* | 1 ± 0.19 | 1.1 ± 0.18 | 1.86 ± 0.27* | 0.04 |
| *Aplnr* | 1 ± 0.21 | 1.24 ± 0.18 | 1.35 ± 0.16 | 0.27 |
| *Bdrkb1* | 1 ± 0.54 | 0.85 ± 0.25 | 0.43 ± 0.15 | 0.16 |
| *Bdrkb2* | NA | | |  |
| *Calca* | 1 ± 0.22 | 1.12 ± 0.24 | 0.88 ± 0.29 | 0.29 |
| *Camk2b* | 1 ± 0.22 | 1.83 ± 0.71 | 1.16 ± 0.3 | 0.56 |
| *Camk2d* | 1 ± 0.26 | 2.11 ± 0.34* | 2.92 ± 0.61** | 0.005 |
| *Camk2g* | 1 ± 0.13 | 1.91 ± 0.16** | 1.81 ± 0.29* | 0.008 |
| *Cat* | 1 ± 0.28 | 2.94 ± 0.87** | 3.3 ± 0.83** | 0.003 |
| *Cav1* | 1 ± 0.2 | 1.63 ± 0.29 | 2.1 ± 0.44* | 0.03 |
| *Ccr5* | 1 ± 0.12 | 2.31 ± 0.65 | 1.35 ± 0.34 | 0.13 |
| *Ccr6* | 1 ± 0.2 | 0.97 ± 0.3 | 0.97 ± 0.36 | 0.56 |
| *Ccr7* | NA | | |  |
| *Cd44* | 1 ± 0.11 | 2.60 ± 0.35** | 2.98 ± 0.73*** | 0.0008 |
| *Chrm3* | 1 ± 0.28 | 1,87 ± 0.48 | 1.27 ± 0.53 | 0,17 |
| *Cspr3* | 1 ± 0.22 | 1.25 ± 0.22 | 2.38 ± 0.45** | 0.01 |
| *Ctnnb1* | 1 ± 0.27 | 2.44 ± 0.59* | 3.18 ± 0.76** | 0.007 |
| *Cx3cr1* | 1 ± 0.18 | 2.79 ± 0.61* | 3.17 ± 0.78* | 0.018 |
| *Cxcl12* | 1 ± 0.12 | 1.72 ± 0.2 | 1.7 ± 0.29 | 0.051 |
| *Cxcr3* | 1 ± 0.21 | 1.35 ± 0.35 | 0.94 ± 0.24 | 0.42 |
| *Cxcr4* | 1 ± 0.43 | 0.76 ± 0.17 | 0.83 ± 0.19 | 0.97 |
| *Edn* | 1 ± 0.13 | 1.16 ± 0.23 | 1.22 ± 0.21 | 0.8 |
| *Ednra* | 1 ± 0.38 | 2.43 ± 0.64* | 3.84 ± 1.08* | 0.026 |
| *Ednrb* | 1 ± 0.18 | 0.68 ± 0.16 | 1.66 ± 0.28^#^ | 0.025 |
| *Egf* | 1 ± 0.17 | 1.13 ± 0.42 | 0.59 ± 0.17 | 0.1 |
| *Esr1* | 1 ± 0.22 | 1.15 ± 0.33 | 1.23 ± 0.19 | 0.95 |
| *F2r* | 1 ± 0.21 | 1.21 ± 0.3 | 1.59 ± 0.43 | 0.11 |
| *F2rl1* | 1 ± 0.21 | 1.21 ± 0.3 | 1.5 ± 0.31 | 0.43 |
| *Fgf2* | 1 ± 0.15 | 0.93 ± 0.22 | 1.58 ± 0.25 | 0.08 |
| *Fprl2* | NA | | |  |
| *Gata4* | 1 ± 0.18 | 1.75 ± 0.25 | 1.88 ± 0.34 | 0.098 |
| *Gsk3b* | 1 ± 0.23 | 1.39 ± 0.3 | 2.11 ± 0.35** | 0.013 |
| *Hdac4* | 1 ± 0.23 | 2.28 ± 0.83 | 3.4 ± 0.79 | 0.085 |
| *Hdac5* | 1 ± 0.23 | 1.39 ± 0.3 | 2.11 ± 0.35** | 0.013 |
| *Hdac6* | 1 ± 0.57 | 1.28 ± 0.28 | 3.39 ± 1.07 | 0.28 |
| *Hdac7* | NA | | |  |
| *Hgf* | 1 ± 0.19 | 1.29 ± 0.1 | 1.7 ± 0.43 | 0.46 |
| *Hif1* | 1 ± 0.24 | 1.18 ± 0.27 | 1.96 ± 0.27* | 0.026 |
| *Hrh1* | 1 ± 0.27 | 0.95 ± 0.17 | 1.1 ± 0.33 | 0.72 |
| *Htr3a* | NA | | |  |
| *Htr4* | NA | | |  |
| *Igf1* | 1 ± 0.16 | 0.82 ± 0.12 | 0.94 ± 0.14 | 0.85 |
| *Igf1r* | 1 ± 0.21 | 2.06 ± 0.4 | 3.87 ± 1.05** | 0.018 |
| *Il10* | NA | | |  |
| *Il17* | 1 ± 0.23 | 1.58 ± 0.28 | 0.64 ± 0.2 | 0.06 |
| *Ins2* | 1 ± 0.26 | 1.49 ± 0.38 | 1.07 ± 0.39 | 0.46 |
| *Jak2* | 1 ± 0.29 | 1.13 ± 0.23 | 1.68 ± 0.36 | 0.45 |
| *Kiss1r* | NA | | |  |
| *Kit* | 1 ± 0.21 | 1.71 ± 0.24 | 1.83 ± 0.31* | 0.047 |
| *Lep* | 1 ± 0.3 | 1.46 ± 0.48 | 0.82 ± 0.22 | 0.3 |
| *Mapk1* | 1 ± 0.35 | 1.18 ± 0.25 | 2.25 ± 0.64 | 0.21 |
| *Mapk10* | 1 ± 0.37 | 1.62 ± 0.77 | 0.46 ± 0.21 | 0.19 |
| *Mapk14* | 1 ± 0.2 | 1.6 ± 0.34 | 1.61 ± 0.31 | 0.7 |
| *Mapk3* | 1 ± 0.12 | 3.23 ± 0.56** | 4.69 ± 1.33*** | 0.002 |
| *Mef2a* | 1 ± 0.2 | 1.52 ± 0.18 | 1.7 ± 0.28 | 0.082 |
| *Mef2c* | 1 ± 0.22 | 2.12 ± 0.42* | 2.47 ± 0.35** | 0.007 |
| *Mef2d* | 1 ± 0.17 | 2.02 ± 0.3* | 2.27 ± 0.45** | 0.007 |
| *Mtor* | 1 ± 0.39 | 3.85 ± 1.34 | 3.5 ± 1.03 | 0.069 |
| *Muc1* | NA | | |  |
| *Myoc* | 1 ± 0.33 | 2.21 ± 0.56* | 3.1 ± 0.91** | 0.018 |
| *Nfkb* | 1 ± 0.46 | 1.47 ± 0.23 | 1.41 ± 0.32 | CHI SQUARE |
| *Nos3* | 1 ± 0.41 | 1.86 ± 0.34* | 2.77 ± 0.78** | 0.016 |
| *Nppa* | 1 ± 0.35 | 17.3 ± 7.23** | 13.06 ± 5.43* | 0.058 |
| *Nppb* | 1 ± 0.12 | 3 ± 0.53** | 3.78 ± 0.87*** | 0.0014 |
| *Nr32c* | 1 ± 0.31 | 1.67 ± 0.68 | 2.22 ± 0.6 | 0.08 |
| *Pik3ca* | 1 ± 0.34 | 1.01 ± 0.2 | 1.56 ± 0.33 | 0.19 |
| *Pik3cg* | 1 ± 0.15 | 0.71 ± 0.05 | 1.1 ± 0.26 | 0.71 |
| *Pten* | 1 ± 0.15 | 0.95 ± 0.12 | 1.21 ± 0.11 | 0.28 |
| *Ren* | NA | | |  |
| *Rps6kb1* | 1 ± 0.17 | 1.38 ± 0.38 | 1.45 ± 0.33 | 0.48 |
| *Ryr2* | 1 ± 0.09 | 1.29 ± 0.14 | 1.5 ± 0.18 | 0.058 |
| *S100a* | 1 ± 0.14 | 0.92 ± 0.18 | 0.69 ± 0.14 | 0.19 |
| *Sod* | 1 ± 0.05 | 0.55 ± 0.15** | 0.63 ± 0.1* | 0.003 |
| *Src* | 1 ± 0.38 | 2.18 ± 0.66 | 2.1 ± 0.6 | 0.28 |
| *Stat3* | 1 ± 0.15 | 4.39 ± 1** | 5.51 ± 1.4*** | 0.0007 |
| *Tgfb1* | 1 ± 0.21 | 2.19 ± 0.34* | 3.32 ± 0.78*** | 0.002 |
| *Tlr2* | 1 ± 0.19 | 1.43 ± 0.3 | 1.12 ± 0.37 | 0.32 |
| *Tlr4* | 1 ± 0.15 | 1.42 ± 0.38 | 1.23 ± 0.29 | 0.76 |
