# Supplementary material for: Axl expression is increased in early stages of left ventricular remodeling in an animal model with pressure-overload
Source: PLoS One. 2019 Jun 10;14(6):e0217926. doi: 10.1371/journal.pone.0217926 (PMC6557565; doi:10.1371/journal.pone.0217926)
Supplement: S1 Methods — (DOCX) [file pone.0217926.s008.docx]

Messenger RNA extraction from LV, LK and aAo was accomplished with Trizol Reagent (Invitrogen, Thermo Fisher) tissue disruption followed with silica column purification following the *mir*Vana miRNA Isolation Kit protocol (Invitrogen, AM1561). Briefly, 50-100mg of LV or LK and 5-7mg of aAo were cut into pieces, resuspended in Trizol (1 ml LV or LK; 0.5 ml aAo) and homogenized with an Omni TH homogenizer (Omni International Inc.). Further processing was performed as indicated in the *mir*Vana miRNA Isolation Kit protocol (Invitrogen, AM1561). We added 1/10 of the volume of miRNA Homogenate Additive to the homogenate and mixed well by inverting the tube several times. The mix was left on ice during 10 min and afterward 1 ml of Acid-Phenol:Chloroform was added and the solution was vortexed between 30-60 s. Samples were later centrifuged at 10000g during 5 min at RT. The upper phase was carefully removed and transferred to a new tube. The transferred volume was annotated and 1.25 volume of absolute ethanol (ACS grade) was added to the tube. The mix was added to a Filter Cartridge and all centrifugation and washes steps were done as indicated in the manufacturer’s protocol. Final elution of RNA was accomplished with elution buffer pre-heated at 95°C. RNA quality and quantity were assessed with a NanoDrop 2000c (Thermo Fisher). RNA integrity was checked with denaturing MOPS-formaldehyde gels.

Total RNA (1μg for LV and LK; 0.5μg for aAo) was retrotranscripted with the High-Capacity cDNA Reverse Transcription Kit (4374966, Thermo Fisher) in the presence of RNAse inhibitors. *Axl*, LV remodelling and fibrosis markers mRNA levels were measured with Real time PCR with the enzyme SYBR Select Master Mix (4472908, Thermo Fisher). The PCR protocol was as follows; 2 min at 50°C/ 10 min at 95°C/ 40 cicles of 15 s at 95°C + 1 min at the Annealing Temperature (aT)/ and a continuous change of Temperature increase at 0.05°C/sec from the aT till 95°C to get the melting curve. A ViiA 7 machine and the ViiA 7 software v1.2.1 (Applied Biosystems) were used for product amplification and analysis. Primers were purchased at IBIAN technologies. All amplifications gave a single melting point in the melting curve, and amplicon size was checked with agarose gels. Detailed primer information can be found in the Table 1 below. Real time PCR results were analyzed with the 2^−ΔΔCt^ method, with normalization of the mRNA levels to the cyclophilin B (*Ppib*) housekeeping gene and to control samples.

**Table 1.** **Primers used for real-time PCR experiments.**

| ***Gene*** | ***NCBIseq*** | ***F_primer (5’-3’)*** | ***R_primer (5’-3’)*** | ***aT*** | ***Asize*** |
| --- | --- | --- | --- | --- | --- |
| αMHC | NM_017239 | GCTGGACATGCTGCTGGTTACC | TGCTCCTCCCGCTGCTTCT | 61.5 | 225 |
| βMHC | NM_017240.2 | GCTGAGGAGGACAAGGTCAACACT | GTCGCTCATCCAACTGCTGCTT | 64 | 205 |
| Axl | NM_001013147 | CCTGCTCTGGCTTCAAGATG | CGTGGGATGTCTGGAAACC | 60 | 208 |
| BNP | NM_031545.1 | GTCCTAGCCAGTCTCCAGAA | GCTTGAACTATGTGCCATCTTG | 60 | 203 |
| Col1a1 | NM_053304.1 | CCCAGCGGTGGTTATGACTT | TCGATCCAGTACTCTCCGCT | 60 | 257 |
| Col3a1 | NM_032085.1 | AGTGGCCATAATGGGGAACG | CAGGGTTTCCATCCCTTCCG | 60 | 94 |
| Fib | NM_019143.2 | CCACCATCACTGGTCTGGAG | GGGTGTGGAAGGGTAACCAG | 60 | 136 |
| Mmp2 | NM_031054.2 | TGGCACCACCGAGGATTATG | CCCACAGTGGACATAGCAGT | 60 | 72 |
| Ppib | NM_022536.2 | AGCGCAATATGAAGGTGCTCT | CTTATCGTTGGCCACGGAGG | 60 | 92 |
| TGFβ1 | NM_021578.2 | CCCCTACATTTGGAGCCTGG | TTGCGACCCACGTAGTAGAC | 60 | 141 |
| Timp1 | NM_053819.1 | CCTAGAGACACGCTAGAGCAG | AGCAACAAGAGGATGCCAGA | 59 | 73 |
| Vim | NM_031140.1 | TGCGGCTGCGAGAAAAATTG | GGTCAAGACGTGCCAGAGAA | 60 | 111 |

An abbreviation of the mRNA analyzed, with the corresponding NCBI Nucleotide sequence code, forward (F_primer) and reverse primers (R_primer) sequences, Annealing Temperature (aT) and amplicon size in base pairs (Asize) are listed.

To assess the hypertrophy pathways activated in this model we used the cardiac hypertrophy R384 plates for rat (CFX384, Bio-Rad, assayed genes in Table 2 below), with 96-well predesigned with cardiac hypertrophy and housekeeping genes and quality control assays. Five μg of total RNA were retrotranscribed with the iScript cDNA Synthesis Kit (1708891, Bio-Rad). The cDNA was diluted to a final 25 ng/μl concentration and gene expression levels were assayed with the SsoAdvanced Universal SYBR Green Supermix (1725272, Bio-Rad) and the plates. Cardiac hypertrophy mRNA levels were normalized with the geometric mean of *Gapdh* and *Hprt1*, as suggested by the manufacturer. Results are shown with the 2^−ΔΔCt^ method.

| ***Gene*** | ***Full name*** | ***NCBIseq*** |
| --- | --- | --- |
| Ace3 | Angiotensin-converting enzyme Angiotensin-converting enzyme, soluble form | NM_001258237 |
| Adm | ADM Adrenomedullin Proadrenomedullin N-20 terminal peptide | NM_012715 |
| Adrb2 | Beta-2 adrenergic receptor | Not Available |
| Ager | Advanced glycosylation end product-specific receptor precursor | NM_053336 |
| Agt | Angiotensinogen Angiotensin-1 Angiotensin-2 Angiotensin-3 | NM_134432 |
| Agtr1a | Type-1A angiotensin II receptor | NM_030985 |
| Agtr2 | Type-2 angiotensin II receptor | NM_012494 |
| Akt1 | RAC-alpha serine/threonine-protein kinase | NM_033230 |
| Akt2 | RAC-beta serine/threonine-protein kinase | Not Available |
| Akt3 | RAC-gamma serine/threonine-protein kinase | NM_031575 |
| Aplnr | Apelin receptor | NM_031349 |
| Ar | Androgen receptor | NM_012502 |
| Bdkrb1 | B1 bradykinin receptor | NM_030851 |
| Bdkrb2 | B2 bradykinin receptor isoform 1 | NM_173100 |
| Calca | Calcitonin | NM_001033956, NM_001033955, NM_017338 |
| Camk2b | Calcium/calmodulin-dependent protein kinase type II subunit beta isoform 2 | NM_001042356, NM_001042354, NM_021739 |
| Camk2d | Calcium/calmodulin-dependent protein kinase type II subunit delta | NM_012519 |
| Camk2g | Calcium/calmodulin-dependent protein kinase type II subunit gamma | Not Available |
| Cat | Catalase | NM_012520 |
| Cav1 | Caveolin-1 alpha isoform | NM_031556,NM_133651 |
| Ccr5 | C-C chemokine receptor type 5 | NM_053960 |
| Ccr6 | C-C chemokine receptor type 6 | Not Available |
| Ccr7 | C-C chemokine receptor type 7 precursor | Not Available |
| Cd44 | CD44 antigen precursor | NM_012924 |
| Chrm3 | Muscarinic acetylcholine receptor M3 | Not Available |
| Csrp3 | Cysteine and glycine-rich protein 3 | NM_057144 |
| Ctnnb1 | Catenin beta-1 | NM_053357 |
| Cx3cr1 | CX3C chemokine receptor 1 | NM_133534 |
| Cxcl12 | Chemokine (C-X-C motif) ligand 12 isoform alpha precursor | NM_001033883, NM_001033882, NM_022177 |
| Cxcr3 | C-X-C chemokine receptor type 3 | NM_053415 |
| Cxcr4 | C-X-C chemokine receptor type 4 | Not Available |
| Edn1 | Endothelin-1 | NM_012548 |
| Ednra | Endothelin-1 receptor | NM_012550 |
| Ednrb | Endothelin B receptor | NM_017333 |
| Egf | Pro-epidermal growth factor precursor | Not Available |
| Esr1 | Estrogen receptor | NM_012689 |
| F2r | Proteinase-activated receptor 1 precursor | NM_012950 |
| F2rl1 | Proteinase-activated receptor 2 | NM_053897 |
| Fgf2 | Fibroblast growth factor 2 | NM_019305 |
| Fpr2l | Protein Fpr2 | Not Available |
| Gapdh | Glyceraldehyde-3-phosphate dehydrogenase | NM_017008 |
| Gata4 | Transcription factor GATA-4 | Not Available |
| gDNA | PrimePCR DNA Contamination Control Assay |  |
| Gsk3b | Glycogen synthase kinase-3 beta | Not Available |
| Hdac4 | Histone deacetylase 4 | Not Available |
| Hdac5 | Histone deacetylase 5 | Not Available |
| Hdac6 | Protein Hdac6 | Not Available |
| Hdac7 | Uncharacterized protein | Not Available |
| Hgf | Hepatocyte growth factor Hepatocyte growth factor alpha chain Hepatocyte growth factor beta chain | NM_017017 |
| Hif1a | Hypoxia-inducible factor 1-alpha | NM_024359 |
| Hprt1 | Hypoxanthine-guanine phosphoribosyltransferase | NM_012583 |
| Hrh1 | Histamine H1 receptor | NM_017018 |
| Htr3a | 5-hydroxytryptamine receptor 3A | NM_024394 |
| Htr4 | 5-hydroxytryptamine receptor 4 | Not Available |
| Igf1 | Insulin-like growth factor I | Not Available |
| Igf1r | Insulin-like growth factor 1 receptor Insulin-like growth factor 1 receptor alpha chain Insulin-like growth factor 1 receptor beta chain | NM_052807 |
| Il10 | Interleukin-10 | NM_012854 |
| Il17a | Interleukin-17A precursor | NM_001106897 |
| Ins2 | Insulin-2 Insulin-2 B chain Insulin-2 A chain | Not Available |
| Jak2 | Tyrosine-protein kinase JAK2 | Not Available |
| Kiss1r | KiSS-1 receptor | NM_023992 |
| Kit | Mast/stem cell growth factor receptor precursor | Not Available |
| Lep | Leptin | NM_013076 |
| Mapk1 | Mitogen-activated protein kinase 1 | NM_053842 |
| Mapk10 | Mitogen-activated protein kinase 10 isoform 1 | NM_012806 |
| Mapk14 | Mitogen-activated protein kinase 14 | NM_031020 |
| Mapk3 | Mitogen-activated protein kinase 3 | NM_017347 |
| Mef2a | Myocyte-specific enhancer factor 2A | Not Available |
| Mef2c | Myocyte enhancer factor 2C | Not Available |
| Mef2d | Myocyte-specific enhancer factor 2D | Not Available |
| Mtor | Serine/threonine-protein kinase mTOR | NM_019906 |
| Muc1 | Mucin-1 precursor | Not Available |
| Myocd | Myocardin | NM_182667 |
| Nfkb1 | Nuclear factor NF-kappa-B p105 subunit Nuclear factor NF-kappa-B p50 subunit | Not Available |
| Nos3 | Nitric oxide synthase, endothelial | NM_021838 |
| Nppa | Natriuretic peptides A Atrial natriuretic factor Auriculin-B Auriculin-A Atriopeptin-1 Atriopeptin-2 Atriopeptin-3 | NM_012612 |
| Nppb | Natriuretic peptides B Brain natriuretic peptide 45 | NM_031545 |
| Nr3c2 | Mineralocorticoid receptor | NM_013131 |
| PCR | PrimePCR Positive Control Assay |  |
| Pik3ca | Phosphatidylinositol-4,5-bisphosphate 3-kinase catalytic subunit alpha isoform | Not Available |
| Pik3cg | Phosphoinositide-3-kinase, catalytic, gamma polypeptide | Not Available |
| Pten | Phosphatidylinositol-3,4,5-trisphosphate 3-phosphatase and dual-specificity protein phosphatase PTEN | NM_031606 |
| Ren | Renin | NM_012642 |
| Rps6kb1 | Ribosomal protein S6 kinase beta-1 | Not Available |
| RQ1 | PrimePCR RNA Quality Assay |  |
| RQ2 | PrimePCR RNA Quality Assay |  |
| RT | PrimePCR Reverse Transcription Control Assay |  |
| Ryr2 | Ryanodine receptor 2 isoform 2 | NM_001191043, NM_032078 |
| S100a1 | Protein S100-A1 | Not Available |
| Sod1 | Superoxide dismutase [Cu-Zn] | NM_017050 |
| Src | Proto-oncogene tyrosine-protein kinase Src | Not Available |
| Stat3 | Signal transducer and activator of transcription 3 | Not Available |
| Tbp | TATA-box-binding protein | NM_001004198 |
| Tgfb1 | Transforming growth factor beta-1 Latency-associated peptide | NM_021578 |
| Tlr2 | Toll-like receptor 2 precursor | Not Available |
| Tlr4 | Toll-like receptor 4 precursor | Not Available |

**Table 2.** Full name and the NCBI sequence of the analyzed mRNA listed in table 2A, as provided in the Bio-Rad file.
