# Supplementary material for: Axl expression is increased in early stages of left ventricular remodeling in an animal model with pressure-overload
Source: PLoS One. 2019 Jun 10;14(6):e0217926. doi: 10.1371/journal.pone.0217926 (PMC6557565; doi:10.1371/journal.pone.0217926)
Supplement: S2 Methods — (DOCX) [file pone.0217926.s009.docx]

Soluble AXL (sAXL) was quantified in plasma samples diluted 1/3 with PBS, with the commercial ELISA Kit for AXL Receptor Tyrosine Kinase in Rat (EKU02655, Biomatik) as indicated in the manufacturer protocol.

Proteins from frozen LV samples were extracted from sham (n=5) and LVH (n=8) and HF (n=10) groups. Samples were homogenized in ice-cold RIPA Buffer (89901, Sigma,) containing Halt Protease Inhibitor Cocktail 100x (87786, Thermo Scientific) and Halt Phosphatase Inhibitor Cocktail 100x (78420, Thermo Scientific) with an Omni TH homogenizer (Omni International Inc.). After one hour rotating at 4°C, samples were centrifuged at 10,000g at 4°C for 30 minutes. Supernatant was collected, and total protein concentration was quantified with the Pierce BCA Protein Assay Kit (23227, Thermo Scientific), relative to a BSA standard curve. Reducing buffer with β-mercaptoethanol was added to extract and heated at 95°C for 5 minutes. Electrophoresis of 30 μg of total protein reduced with β-mercaptoethanol was developed in NuPAGE 4-12% Bis-Tris Gel and transferred onto a nitrocellulose membrane with the iBlot Dry Blotting System (Invitrogen) and proper transfer was checked by Ponceau staining. Membranes were blocked for 1 hour with blocking buffer (PBS, 0.1% Tween 20, and 5% milk) and incubated overnight at 4°C with the polyclonal anti-AXL antibody (ab-72069, abcam; 1/500 dilution). Next day, membranes were washed and incubated 1 hour with a goat anti-rabbit secondary antibody (#31460, Thermo Scientific,1/3000 dilution). Imaging was performed in the Image Quant LAS4000 (GE Healthcare, OH, USA) using the SuperSignal West Pico Chemiluminescent Substrate (Thermo Scientific). Bands were quantified by Image Lab Software (Biorad). Results were normalised with band quantification of the Ponceau staining and are given in relative units (R.U.).
