# Supplementary material for: Axl expression is increased in early stages of left ventricular remodeling in an animal model with pressure-overload
Source: PLoS One. 2019 Jun 10;14(6):e0217926. doi: 10.1371/journal.pone.0217926 (PMC6557565; doi:10.1371/journal.pone.0217926)
Supplement: S3 Methods — (DOCX) [file pone.0217926.s010.docx]

Paraffin embedded apex slides were deparaffinated and hydrated with a battery of xylene (1417690314, Panreac), and alcohols (20821.365, VWR) with increasing concentrations of water. Antigen retrieval was achieved with a 10 mM citrate bath (C7254, Sigma) at 85°C during 40 minutes. Endogenous peroxidase activity was blocked as indicated with the Dako Envision kit (K4011, Dako) and slides were incubated during 30 min with a blocking buffer (PBS, 0.5% Triton X-100, 3% goat serum) previous to an overnight incubation with polyclonal rabbit Axl antibody (PA5-23254, ThermoFisher) diluted 1/200. Next day, slides were treated as indicated in the Dako Envision kit. The preparations were counterstained with hematoxylin of Gill (GHS132, Sigma) and dehydrated with an increasing alcohol concentration battery and xylenes and mounted with DPX (1.00579.0500, Merck). Pictures were taken with an Olympus BX51 microscope, an Olympus DP71 camara and the Cell Imaging Software.
